# Differences between peptide profiles of extensive hydrolysates and their influence on functionality for the management of cow's milk allergy: A short review

**DOI:** 10.3389/falgy.2022.950609

**Published:** 2022-09-05

**Authors:** Anne Goh, Leilani Muhardi, Adli Ali, Woei Kang Liew, Elizabeth Estrada-Reyes, Benjamin Zepeda-Ortega, Urszula Kudla, R. J. Joost van Neerven, Laurien H. Ulfman, Tim T. Lambers, John O. Warner

**Affiliations:** ^1^ Department of Paediatrics, KK Women’s and Children’s Hospital, Singapore, Singapore; ^2^ Medical Affairs, Friesland Campina AMEA, Singapore, Singapore; ^3^ Department of Paediatrics, Universiti Kebangsaan Malaysia Medical Center, Bangi, Malaysia; ^4^ Paediatric Allergy Immunology Rheumatology Centre, Mount Elizabeth Novena Specialist Centre, Singapore, Singapore; ^5^ Department of Pediatrics, Hospital Angeles Metropolitano, Mexico, Mexico; ^6^ Department of Pediatrics, Angeles Lomas Hospital Huixquilucan Mexican State, Mexico, Mexico; ^7^ R&D, FrieslandCampina, Amersfoort, the Netherlands; ^8^ Cell Biology and Immunology, Wageningen University, Wageningen, the Netherlands; ^9^ National Heart and Lung Institute, Imperial College, London, United Kingdom; ^10^ Departement Pediatrics and Child Health, University of Cape Town, Cape Town, South Africa

**Keywords:** peptide profiling, extensively hydrolysed formula, cow’s milk protein allergy, management, efficacy

## Abstract

Extensively hydrolyzed formulas (eHFs) are recommended for the dietary management of cow's milk protein allergy (CMPA) in non-exclusively breastfed infants. Studies show that peptide profiles differ between eHFs. This short review aims to highlight the variability in peptides and their ability to influence allergenicity and possibly the induction of tolerance by different eHFs. The differences between eHFs are determined by the source of the protein fraction (casein or whey), peptide size-distribution profile and residual *β*-lactoglobulin which is the most immunogenic and allergenic protein in bovine milk for human infants as it is not present in human breastmilk. These differences occur from the hydrolyzation process which result in variable IgE reactivity against cow's milk allergen epitopes by subjects with CMPA and differences in the Th1, Th2 and pro-inflammatory cytokine responses elicited. They also have different effects on gut barrier integrity. Results suggest that one particular eHF-casein had the least allergenic potential due to its low residual allergenic epitope content and demonstrated the greatest effect on restoring gut barrier integrity by its effects on mucin 5AC, occludin and Zona Occludens-1 in human enterocytes. It also increased the production of the tolerogenic cytokines Il-10 and IFN-*γ*. In addition, recent studies documented promising effects of optional functional ingredients such as pre-, pro- and synbiotics on the management of cow's milk allergy and induction of tolerance, in part *via* the induction of the production of short chain fatty acids. This review highlights differences in the residual allergenicity, peptide size distribution, presence of optional functional ingredients and overall functionality of several well-characterized eHFs which can impact the management of CMPA and the ability to induce immune tolerance to cow's milk protein.

## Background

The incidence of food allergy among young children has increased in the last two decades (1). The prevalence of cow's milk protein allergy (CMPA) has been reported between 1.4%–3.8% of infants (1). The prevalence varies due to the methods used for diagnosis and reporting across studies. Based on the symptoms and the presence of immunoglobulin E (IgE), CMPA is conventionally classified as IgE - mediated allergy, non-IgE mediated allergy, or mixed (both IgE and non-IgE mediated) (1). The diverse range of symptoms involving many different organ systems can further influence the reported prevalence (2). There also has been an alteration in the natural history of cow's milk allergy resulting in a higher risk of persistence into later childhood (3, 4).

In children with CMPA who cannot be exclusively breastfed, extensively hydrolyzed milk protein formulas (eHF) have been advocated as the first choice in the dietary management of CMPA in many international guidelines including the recent WAO recommendation (1, 5, 6). In the situation where partial breastfeeding is provided, it is not advisable for lactating mothers to have a milk-protein-free diet (1).

However, not all commercially available eHFs have the same hydrolyzation process and subsequent peptide profiles can affect their efficacy for the dietary management of CMPA (7, 8). Differences among eHFs in efficacy can be due to differences in the source of the protein fraction (casein (C) or whey (W)), the peptide size-distribution profile resulting from the hydrolyzation process, and/or the presence of other functional ingredients such as probiotics, prebiotics, and Long-Chain Poly-Unsaturated Fatty Acids (LCPUFAs) (8–11).

**Figure 1:**
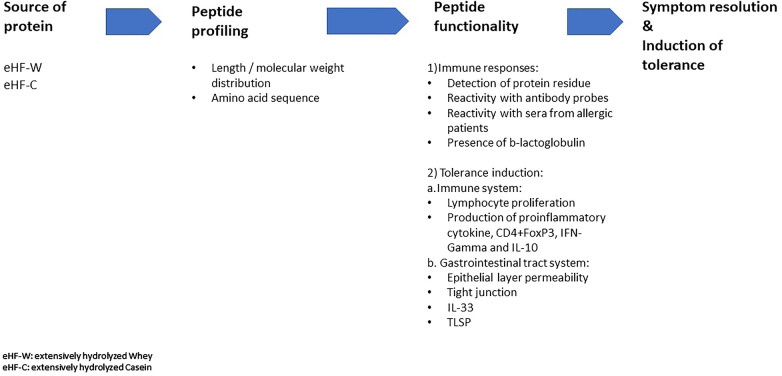
Distinct features of extensive hydrolysate and the impact on its effectiveness.

Several recent publications have suggested distinct features among commercially available eHFs by which their peptide profiling and functionality affect immune responses and their potential for immune tolerance in CMPA (8–10, 12). This short review aims to provide an overview of the distinct features of peptides in various commercially available eHFs, such as the source of the protein fraction, molecular weight distribution, and the T-cell activating capacity of the residual peptides in the eHF. These distinct features could affect their effectiveness in the management of CMPA (Figure 1).

## Peptide profiling

The American Academy of Pediatrics (AAP) in 2000 defined an eHF as a formula containing peptides with a molecular weight <3 kDa (13). However, as some allergic reactions were still reported in selected cases using eHF, the British Society for Allergy and Clinical Immunology (BSACI) guidelines in 2014 suggested that an eHF is one that contains a greater percentage of peptides <1 kDa with less than 5% of peptides >3 kDa for the nutritional management of CMPA (14). Moreover, it is a prerequisite to document hypo-allergenicity of the eHF, clinically defined by AAP as reduced allergenicity or reduced ability to stimulate an IgE response and induce IgE-mediated reactions (13).

More recent research shows that molecular weight is not the only factor determining the possibility of allergic reaction. For IgE-cross linking, to induce mast cell degranulation, the peptides need to be larger than 3 kDa or a minimum length of 30 amino acids. For a protein to have allergenic properties, it has to contain at least two IgE binding sites and enable cross linking of Fc*ε*RI on the cell membrane of basophils and mast cells (15). In intact proteins, a B cell epitope needs a solvent exposed area of around 500 Å2 (16). In contrast, T-cell epitopes that are presented in the context of major histocompatibility complex (MHC) class II are only 12–18 amino acids (AA) long, although due to the open end of the binding site some overhang is possible, allowing peptides of up to 25 amino acids to be presented to the T cell (17, 18). Thus, hypothetically, if a hydrolysate does not contain peptides of at least 12 AAs, these hydrolysates cannot activate T cells. Hydrolysates that do contain such peptides can induce T cell activation. A hydrolysate containing peptides of between 12 and 30 AA can therefore efficiently activate CD4+ T lymphocytes but cannot induce sensitization as they are too small to contain a B-cell epitope.

Upon T cell activation, B cells will class switch their immunoglobulin production under the influence of T cell derived cytokines. T cell derived Interleukin (IL)-4 production will direct the process of Ig class switching towards IgE causing sensitization and subsequent allergy. Whereas IL-10 promotes the production of immunoglobulin G4 (IgG4) which is involved in the process of immune tolerance. Hence besides reducing allergenicity of an eHF based on peptide molecular weight and length distribution, it is also important to determine the functionality of the residual peptides which can contribute to their effectiveness in reducing allergic responses as well as the induction of tolerance in the infants and children with CMPA (8, 9).

An attempt to further distinguish the distribution of peptides is through peptidomics, which is a technology that has found its application in many research areas including food sciences due to the rapid development of mass spectrometry-based methodologies (10). Molecular weight determination and methods to determine peptide mass and peptide length distribution profiles do not deliver peptide sequence information, while peptidomics enables the identification and relative quantification of multiple peptides simultaneously. In an analytical study, combinations of peptidomics and multivariate clustering analyses were applied to compare peptide profiles of different eHFs.

Even though eHF-C based formulas have relatively similar peptide coverage, they have distinct clustering profiles (Table 1). Furthermore, the eHF-W had completely different clusters from those of eHF-C which further illustrate the need to distinct the feature of specific eHFs.

**Table 1:** Peptide profile.

	Peptide coverage (10)	Hierarchical clustering (10)	Less than 5% peptides with molecular weight between 3 and 5 kDA (8)
eHF-C-1	*α*S1-casein, αS2-casein, *β*-casein, *κ*-casein, β-lactoglobulin	Cluster 1	+
eHF-C-2	αS1-casein, αS2-casein, β-casein, κ-casein, β-lactoglobulin	Cluster 2	+
eHF-C-3	αS1-casein, αS2-casein, β-casein, κ-casein, β-lactoglobulin	Cluster 1	+
eHF-W-1	αS1-casein, αS2-casein, β-casein, κ-casein, α-lactalbumin, β-lactoglobulin	Cluster 3	+

eHF-C-1, extensive hydrolyzed casein-1 (Nutramigen); eHF-C-2, extensive hydrolyzed casein (Frisolac AC); eHF-C-3, extensive hydrolyzed casein (Similac Alimentum); eHF-W, extensive hydrolyzed whey-1 (Nutrilon Pepti); the number in the bracket refers to the reference.

## Peptide allergenicity

Although demonstrating hypo-allergenicity of eHF in sufficiently powered clinical studies is a prerequisite, in-vitro studies using sera from allergic patients could provide additional information. In an in-vitro study, 10 cow's milk formulas were analyzed in a blinded manner regarding their biochemical and immunological characteristics. The formulas consisted of whole milk, partially hydrolyzed whey with/without casein, eHF whey (eHF-W) and casein (eHF-C) formulas as well as amino acid formulas. Protein, peptide and amino acid contents were determined by measuring protein nitrogen. The allergenic activity of the samples was measured using rat basophil leukemia assays as well as lymphocyte proliferation assays and analysis of cytokine levels from the supernatants. Using a RAST-based assay with sera from cow's milk allergic patients, IgE reactivity towards *α*-lactalbumin and *β*-lactoglobulin were found not surprisingly in the whole milk formulas. IgE reactivity was found also with the partially hydrolyzed formula and the eHF-W and one of the eHF-C formulas. The 2 remaining eHF-C and amino acid formula showed low allergenic and low pro-inflammatory properties (9) (Tables 2, 3). Distinct reactivity towards cow's milk (CM) antibodies was found in partially hydrolyzed as well as some eHFs as CM epitopes may remain depending on the hydrolyzation process (8, 9).

**Table 2:** Hydrolysates, its IgE reactivity and the presence of α-lactalbumin and β-lactoglobulin.

	Detection of protein in formula (9)	Reactivity with antibody probes (9)	IgE reactivity with sera from allergic patients (9)	Other allergenic activity^a^ (9)	β-lactoglobulin allergenicity (8)
eHF-C-1	−	−	−	−	−
eHF-C-2	−	−	−	−	−
eHF-C-3	−	−	+	−	−
eHF-W-2	−	+	+	+	−

eHF-C-1, extensive hydrolyzed casein-1 (Nutramigen); eHF-C-2, extensive hydrolyzed casein (Frisolac AC); eHF-C-3, extensive hydrolyzed casein (Similac Alimentum); eHF-W-2, extensive hydrolyzed whey (Alfare), +, detected; −, not detected.

^a^ Other allergenic activity which includes basophil activation and T-cell reactivity. The detection of protein in formula is defined by SDS-Page and Coomassie Brilliant Blue Staining. The number in the bracket refers to the reference.

**Table 3:** Hydrolysates and its effect on lymphocyte proliferation and pro-inflammatory cytokines (9).

	Lymphocyte proliferation	Induction of IFN-*γ*	Induction of IL-13
eHF-C-1	+	−	−
eHF-C-2	++	++	−
eHF-C-3	++	+	+
eHF-W-2	+	+	++
AAF	+	+	+

eHF-C-1, extensive hydrolyzed casein-1 (Nutramigen); eHF-C-2, extensive hydrolyzed casein (Frisolac AC); eHF-C-3, extensive hydrolyzed casein (Similac Alimentum); eHF-W-2, extensive hydrolyzed whey (Alfare); amino acid formula (AAF) ++, Strong stimulation; +, Stimulation; −, minimal or no effect. The number in the bracket refers to the reference.

A recent publication highlighted the importance for not relying exclusively on the peptide size to demonstrate hypo-allergenicity in 4 partially hydrolyzed whey formulas. The researchers used size exclusion chromatography to characterize the peptide molecular weight and a rat basophil degranulation assay to assess the relative level of beta-lactoglobulin allergenicity and a preclinical model of oral tolerance induction to test prevention of allergic sensitization. They found that peptide size was not necessarily associated with allergenicity reduction *in vitro* nor oral tolerance induction *in vivo* as measured by IgE level. Some of the partially hydrolyzed formulas with low peptide molecular weight had high residual beta-lactoglobulin which increased their allergenicity. The authors concluded that not all partially hydrolyzed formulas with the same peptide size distribution decreased allergenicity or had similar ability to induce oral tolerance (19).

## Peptides and immune tolerance

Although most children with CPMA outgrow the symptoms at the ages of 3–4 years, there is also evidence that cow's milk allergy is persisting to an older age, especially in children with associated atopic diseases such as asthma, atopic dermatitis and allergic rhinoconjunctivitis. Hence there is great interest in whether induction of tolerance can be accelerated when managing such patients (1, 3, 20). In a study assessing multiple formulas, two eHFs (eHF-C-2 and eHF-C-3) induced high levels of the Th1 cytokine IFN-*γ* but all 4 formulas had low IL-10 profiles (Table 3). eHF-C-1 did not induce any relevant levels of Th1, Th2 or pro-inflammatory cytokines (Tables 2, 3) (9). Although, it is not clear whether these characteristics alone will influence immune tolerance acquisition, they may at least to a certain extent, contribute to the overall efficacy of the formula to induce tolerance.

Successful allergen immunotherapy (AIT) is achieved by inducing a shift of type 2 immune responses toward a type 1 through an increase in regulatory T (Treg) and regulatory B (Breg) cells and IL-10, with lower IgE production in favour of higher levels of IgG4 antibodies. Current allergen immunotherapy approaches depend on the administration of intact allergens with the incumbent risk of serious side effects. Evolving new modalities include using short and long contiguous overlapping peptides (COPs) targeting dominant T cell epitopes of major allergens in place of intact allergens for AIT. This technique preserves the relevant peptides for T cell recognition but lacks the conformation of the whole protein which prevents IgE binding in the surface of mast cells and basophils, rendering it a safer technique for AIT. Selection of the correct sequence and optimizing the length of the peptide is critical to safety, success, and cost of peptide AIT (21, 22). The aims of AIT for induction of tolerance are, ideally, to (1) induce immunological tolerance by administering a preparation that limits the risk of cross-linking IgE and hence anaphylaxis; (2) induce long term tolerance; (3) reduce the levels of Th2 responses specific for the allergen; (4) increase levels of Foxp3 + Tregs and IL-10-secreting Tr1 cells responding to the allergen; (5) increase the ratio of IgG4:IgE- secreting B cells so as to increase levels of blocking antibodies.

Given the observation that some eHFs have immune tolerizing effects, employing peptidomics on milk protein to characterise T-cell epitopes that can modify cellular immune responses rather than binding of IgE, could be a fruitful research endeavour . For example, therapeutic peptides have been developed for immunotherapy against Japanese cedar pollinosis. Oral administration of one predominant peptide or a 3-linked T cell epitope peptide induced immune tolerance in a mouse model (23) and in humans (24). This novel concept has not been used in food allergy for the induction of tolerance. Given that there are already eHFs available for the management of CMPA, the use of peptidomics and studying the immunological characteristics of the hydrolysates from these eHFs could be applied to peptide immunotherapy in accelerating immune tolerance.

One of the mechanisms by which Tregs (CD4 + CD25+) may enhance the process of tolerance induction is *via* the production of the suppressive cytokine IL-10. Gut barrier dysfunction, leading to an enhanced epithelial permeability and decreased mucus thickness, increases antigen uptake and promotes Th2 type allergic responses by activating type 2 innate lymphoid cells (ILC2s), mast cells, basophils and dendritic cells. Epithelial-derived cytokines, including thymic stromal lymphopoietin (TSLP) and IL-33 have a pivotal role in the development of allergic response at the gut barrier surface which has been linked to the development of food allergy (20, 25).

Recent studies, in both humans and mouse models, have implicated thymic stromal lymphopoietin (TSLP) in the development and progression of allergic diseases as one of the cytokines that is involved in driving allergic inflammatory responses (26). Interleukin-33 (IL-33) is a member of the IL-1 cytokine family that has been widely studied for its dichotomous functions in homeostasis and inflammation (27). It is released by the gut epithelial and endothelial cells in response to cell injury, such as when exposed to proteolytic activity, as an alarmin to initiate the innate immune response. In addition, IL-33 is also an important mediator for the secretion of Th2 related cytokines such as IL-4, IL-5 and IL-13 (27).

It is well established that epithelial cells in the lung respond to allergens by the production of cytokines including IL-33 and TSLP, as well as several other alarmins that drive Th2 immunity (25, 28). Likewise, allergic sensitization to food allergens may occur through the skin (29, 30) and involves the induction of TSLP production by keratinocytes (31).

In an ovalbumin-induced food allergy model, TSLP-induced food allergy was dependent on the presence of IL-33, but IL-33 driven allergy was independent of TSLP (32). A similar role for IL-33 (and TSLP) was shown in a peanut allergy model (33). Notably, a mix of anti-IL-33, anti-TSLP and anti-IL-25 prevented egg allergy induction and suppressed ongoing disease (34).

Although information of direct induction of the production of IL-33 and TSLP by food allergens is sparse to date (35–38), several food allergens display proteolytic activity, and the role of these cytokines in the development food allergies is well established. In addition, food-associated toxins like the mycotoxin deoxynivalenol can also activate the IL-33 and TSLP production by intestinal epithelial cells.

Paparo et al. (35) took 5 formulas which were used for the dietary treatment of CMPA, namely eHF-W, eHF-C, hydrolyzed rice formula (HRF), soy formula (SF) and amino acid formula (AAF), and assessed their effect on epithelial layer permeability and tight junction proteins, mucin 5AC, IL-33 and TSLP in human enterocytes in an in-vitro study. They also looked at Th1/Th2 cytokine response and Treg activation on peripheral blood mononuclear cells from IgE-mediated cow's milk allergic infants. They found that eHF-C derived protein fraction positively modulated the expression of gut barrier components such as mucin 5AC, occludin and Zona Occludens (ZO)-1 in human enterocytes. They also found that only the eHF-C derived protein fraction elicited an increase of the tolerogenic cytokines production, Il-10, IFN-*γ*, and activated CD4 + Foxp3+ Treg through de-methylation of CpG sequences in the Foxp3 gene resulting in up-regulation of gene product production. Though the SF was able to stimulate the expression of occludin only, none of the other formulas were able to produce the same effect on epigenetic modulation compared with eHF-C. Given this activity of the eHF-C formula, it was speculated that using the specific eHF-C employed in the study could accelerate immune tolerance acquisition in children with CMPA (Table 4).

**Table 4:** Hydrolysates and their effects on tolerogenic pathways (35).

	Epithelial layer permeability	tight junction proteins	IL-33	thymic stromal lymphopoietin (TSLP)	IL-10 production	activated CD4 + FoxP3+
eHF-C	+++	+++	−	−	+++	+++
eHF-W	+	++	−−	−−−	++	++
HRF	++	+	−−−	−−−	++	+
SF	+++	++	−−−	−	++	+
AAF	+	+	−−	−	++	+

eHF-C, extensive hydrolyzed casein; eHF-W, extensive hydrolyzed whey; HRF, hydrolyzed rice formula; SF, soy formula; and AAF, Amino Acid Formula (no brand names were provided in this study); +, positive effect (i.e. decreased permeability; number of signs reflecting the magnitude of the effects). –, negative effect (i.e. alarmins production; number of signs reflecting the magnitude of the effects). The number in the bracket refers to the reference.

## Role of optional ingredients: pre-, pro- and synbiotics

There is a growing interest in the potential role of the gut microbiota in the development of allergic disease. Several studies have shown that an altered gut microbiota, or dysbiosis, occurs in allergic infants compared to healthy infants, including those with CMPA (39, 40). It has been demonstrated that allergic infants have low levels of *Bifidobacteria* and *Lactobacilli* in their gut microbiota compared to healthy infants (41). Hence the addition of pre-, pro- and synbiotics could influence the composition of the gut microbiota towards a more “normal” or healthy profile. A study on partially hydrolyzed formula supplemented with short chain galacto-oligosaccharide(scGOS) and long chain fructo-oligosaccharide (lcFOS) resulted in a gut microbiota more similar to breastfed infants as compared to those fed standard cow's milk formula (42). Two earlier studies using mouse models also showed a promising effect on induction of tolerance using whey or partially hydrolyzed whey with rather than without a combination of oligosaccharides (scGOS/lcFOS/pAOS) (43, 44).

In the management of CMPA, it seems logical to consider the addition of probiotics or synbiotics to the eHF in accelerating the acquisition of tolerance to cow's milk given the role of the microbiota and known immunomodulatory activity of pro-, pre and synbiotics. Previously, this was comprehensively reviewed by Fox et al (45). A randomized controlled trial of eHF-C with or without the probiotic *Lactobacillus rhamnosus GG* (LGG) in 55 challenged proven CM allergic infants showed accelerated development of tolerance to cow's milk protein in the group receiving the probiotic supplemented eHF-C compared to eHF-C alone when re-challenged 6 and 12 months later (46). In a larger prospective study 260 children with CMPA aged 1–12 months were allocated to 5 groups based on the formula used for dietary management of CMPA: eHF-C, eHF-C with LGG, hydrolyzed rice formula, soy formula or amino acid formula. This study confirmed that there was an accelerated acquisition of tolerance by eHF-C which was enhanced with the addition of LGG as compared to other tested formulas (47).

However, the study by Hol et al (48) which randomized 119 CM allergic infants to receive another type of eHF-C with and without the addition of probiotics, *Lactobacillus casei* CLR431 and *Bifidobacterium lactis* BB12 revealed that there was no difference between groups in the number of infants who achieved tolerance at the end of 6 months and 12 months when re-challenged to cow's milk. This study showed that the addition of probiotics did not accelerate the acquisition of tolerance to cow's milk with this particular eHF-C formula. Interestingly, the percentage of CMPA infants who develop tolerance measured after 12 month of consumption was similar in both studies; 77% and 81% for the eHF-C with or without probiotics respectively (48) vs. 81% for an e-HF-C with LGG (46) [i.e. comparing the data from eHF-C + LGG to eHF-C only (48)].

In another recently published study, 200 infants suspected to have CMPA based on cow's milk-related symptom score (CoMiSS) were randomized to receive an eHF-W with and without the addition of prebiotics in the form of human milk oligosaccharides (2′FL and LNnT). The researchers found no difference in the resolution of cow's milk associated symptoms between groups. This study suggests that the addition of human milk oligosaccharides to this particular eHF-W did not accelerate the resolution of cow's milk related symptoms (49).

A study which recruited 71 infants with suspected non-IgE mediated CMPA were randomized to receive AAF with and without synbiotics (prebiotic blend of chicory-derived neutral oligofructose and long chain inulin and probiotic strain *Bifidobacterium breve* M-16V). The researcher’s main aim was to investigate the modification of the gut microbiome with the symbiotic supplemented formula, which was compared to healthy age-matched controls. They found that there was an increase in *Bifidobacterium* and a reduction in the *Eubacterium rectale/Clostridium coccoides* percentage in the stool at the end of 8 weeks which reflected a microbiome that was closer to the healthy controls. The study was not designed to look at overall effects and thus the clinical outcomes remain to be established and could not be identified in the publication (50). A subsequent prospective, randomized double-blind, controlled study had 169 challenge confirmed CM allergic infants and randomized them to receive AAF with/without synbiotics (prebiotic blend of chicory-derived neutral oligofructose and long chain inulin and probiotic strain *Bifidobacterium breve* M-16V). The researchers found no difference in the induction of tolerance between groups at 12 and 24 months on re-challenge to cow's milk demonstrating that the addition of synbiotics to the AAF did not accelerate the induction of tolerance (51).

The differences observed between these studies can to a large extent be explained by the use of different formulas including protein source (different hydrolysates and/or amino acids), different optional ingredients (predominantly pro-, pre- and synbiotics) and differences in CMPA subjects enrolled in the study. This again highlights that there may be inherent differences even between eHFs and their ability to induce tolerance regardless of the addition of pro-, pre- or synbiotics and results from one eHF study cannot be generalized for other eHFs. The fact that the overall formula composition, hydrolysate peptide profile and size distribution of eHFs differ considerably is an important point when considering clinical effectiveness, especially in relation to induction of tolerance. Thus, although eHFs are often considered as identical, these results underline that specific eHFs should be considered separately and that clinical results from one formula cannot be generalized to other formula.

## Limitation of the review

This review was conducted non-systematically based on unstructured search terms for publications in the area of milk protein hydrolysates for the dietary management of CMA published in the last 20 years (2002–2022). There were no inclusion/exclusion criteria set *a priori*, although both clinical and non-clinical mechanistic or analytical studies have been included. Further, studies were added based on information in the cross-references and for which the authors agreed were relevant to the review topic. There were only a limited number of retrieved and relevant literature which studied different types of eHFs.

## Conclusion

Based on the limited available literature on detailed characterization of eHFs, each of the eHF described have distinct peptide profiles which can impact residual IgE binding and T-cell tolerizing capacity. These differences, with or without the presence of optional functional ingredients like pre-, pro- and synbiotics illustrate the importance of characterizing each commercially available eHF. Thus, although eHFs are often considered as identical, these results underline that all eHFs should be considered separately even those with similar sources of protein fraction. The clinical results from one formula can therefore not be generalized to another formula.

## Impact statement

Even though extensive hydrolyzed formulas (eHF) are the first choice for dietary management of cow's milk protein allergy (CMPA) in infants and children, clinical efficacy of commercially available formulas for dietary management of CMPA differ. This short review reported differences in peptide profiling (peptide length, molecular weight distribution and amino acid sequences) which influences tolerance induction and residual allergenic potential. The addition of functional ingredients (pre-, pro-, synbiotics and long-chain polyunsaturated fatty acids) can further facilitate the development of tolerance acquisition to CMPA. Thus, efficacy studies to show an association between specific peptide profiles, their effect on elicited immune responses, gut barrier integrity and tolerogenic cytokines, with symptom resolution and tolerance induction, are warranted for each specific eHF.

## Social media statement

Extensively hydrolyzed formula (eHF) efficacy in cow's milk protein allergic infants.
